# Fast and Robust Real-Time Estimation of Respiratory Rate from Photoplethysmography

**DOI:** 10.3390/s16091494

**Published:** 2016-09-14

**Authors:** Hodam Kim, Jeong-Youn Kim, Chang-Hwan Im

**Affiliations:** Department of Biomedical Engineering, Hanyang University, Seoul 04763, Korea; rhg910907@hanmail.net (H.K.); jy.kim@bme.hanyang.ac.kr (J.-Y.K.)

**Keywords:** respiratory rate, photoplethysmography, adaptive IIR notch filter

## Abstract

Respiratory rate (RR) is a useful vital sign that can not only provide auxiliary information on physiological changes within the human body, but also indicate early symptoms of various diseases. Recently, methods for the estimation of RR from photoplethysmography (PPG) have attracted increased interest, because PPG can be readily recorded using wearable sensors such as smart watches and smart bands. In the present study, we propose a new method for the fast and robust real-time estimation of RR using an adaptive infinite impulse response (IIR) notch filter, which has not yet been applied to the PPG-based estimation of RR. In our offline simulation study, the performance of the proposed method was compared to that of recently developed RR estimation methods called an adaptive lattice-type RR estimator and a Smart Fusion. The results of the simulation study show that the proposed method could not only estimate RR more quickly and more accurately than the conventional methods, but also is most suitable for online RR monitoring systems, as it does not use any overlapping moving windows that require increased computational costs. In order to demonstrate the practical applicability of the proposed method, an online RR estimation system was implemented.

## 1. Introduction

Respiratory rate (RR), or frequency of breathing, is an essential vital sign that can be used in a variety of clinical and human–computer interaction (HCI) applications. In particular, the precise estimation of RR is of great value, since RR is an auxiliary indicator of potential respiratory dysfunctions associated with various diseases, such as sleep apnea [1] and chronic obstructive pulmonary disease [2]. In addition, continuous monitoring of RR is becoming important in recent HCI applications, as it can be used to monitor changes in emotional arousal during a specific task and/or to present respiration-related biofeedback during meditation or slowed respiration training [3]. 

RR can be precisely measured using capnography, which is the monitoring of the CO_2_ concentration in the respiratory gases; however, this device is not feasible in mobile or wearable forms, because it requires additional equipment, such as a mask or a nasal cannula [4]. Instead, a respiration belt, which measures the changes in thoracic or abdominal circumference, has been frequently used to measure RR in wearable applications [5]. Recently, other techniques for the estimation of RR using wearable sensors have been also developed, such as accelerometers, photoplethysmography (PPG), and electrocardiography (ECG) [6,7]. Among them, PPG has been the most widely studied, as it can be readily recorded using wearable optical sensors attached to a fingertip, wrist, earlobe, or forehead. Although PPG is generally used to measure heart rate (HR) and its variability over time, it also includes a respiration-related component due to the changes in the arterial blood pressure and peripheral venous pressure during respiration [8]. 

A variety of techniques have been proposed to estimate RR from PPG, such as digital filters [9], neural networks [10], wavelet decomposition [11,12], Fourier transform [13], autoregression [14,15], complex demodulation [4], principle component analysis (PCA) [16], independent component analysis (ICA) [17], empirical mode decomposition (EMD) [18], and smart fusion [19]. Among these, PCA and ICA are not appropriate methods for the real-time estimation of RR. Other techniques besides PCA and ICA require the use of overlapping moving windows, which degrades the overall computational efficiency and thus makes real-time applications requiring fast and seamless estimation of RR difficult [20]. Recently, Park et al. proposed an adaptive lattice-type respiratory rate estimator (ALRE) as an alternative technique for the real-time estimation of RR without the need for moving windows [20]; however, their technique required a relatively long convergence time to assure the precise estimation of RR [20]. 

In the present study, we proposed a new method for the real-time estimation of RR from PPG based on frequency estimation. We used an adaptive infinite impulse response (IIR) filter to facilitate faster real-time estimation of RR without the need for moving windows. To the best of our knowledge, the adaptive IIR notch filter has never been applied to the PPG-based estimation of RR, although similar adaptive filters have been applied to RR estimation from a non-contact-type respiration sensor [21]. We verified the performance and practicality of the proposed method, which will hereafter be called Adaptive Infinite Impulse response filter-based Respiratory rate Estimator (AIIRE), not only by applying it to an offline PPG database, but also by implementing an online RR estimation system.

## 2. Methods

### 2.1. Adaptive IIR Notch Filter

To estimate RR from PPG, we applied an adaptive IIR notch filter that can estimate both fundamental and harmonic frequencies [22]. The transfer function of the second-order IIR notch filter is defined as

(1) $$H\left(z\right)=\frac{1-2\cos\left(\theta \right)z^{-1}+z^{-2}}{1-2r\cos\left(\theta \right)z^{-1}+r^{2}z^{-2}}$$

where θ∈(0,π) is the notch frequency and *r* is a parameter that controls the bandwidth of the notch. The filter output, *y*[*n*], for an input signal, *x*[*n*], is then determined as

(2) $$\begin{array}{cc} y\left[n\right]=x\left[n\right] & -2\cos\left(\theta \right)x\left[n-1\right]+x\left[n-2\right]\\ & +2r\cos\left(\theta \right)y\left[n-1\right]-r^{2}y\left[n-2\right] \end{array}$$

The frequency corresponding to *θ* (in radians) is *f* = *θf_s_*/2π, where *f_s_* is the sampling frequency in hertz [22]. For the adaptive process, we need to set the initial *θ* that corresponds to the fundamental frequency, which represents a frequency with the highest power in the power spectrum. Fast Fourier transform (FFT) was used to evaluate the amplitude spectrum. The filter parameter *θ* was then updated using the normalized least mean squares (LMS) algorithm, described by the following equations:

(3) $$\theta \left[n+1\right]=\theta \left[n\right]-2\mu \left[n\right]y\left[n\right]\frac{\partial y\left[n\right]}{\partial \theta \left[n\right]}$$

(4) $$\mu \left[n\right]=\frac{constant}{\hat{P}\left[n\right]}$$

where *μ*[*n*] is the step-size for the adaptive process, and $\hat{P}\left[n\right]$ is an estimated signal power at an instant *n*, which is approximated as $\hat{P}\left[n\right]=\left(1/L\right)\overset{L-1}{\underset{l=0}{\sum }}{\left|x\left[n-l\right]\right|}^{2}.$ To reduce the computational burden, we used the following form containing only two-point data: $\hat{P}\left[n\right]=\hat{P}\left[n-1\right]+({\left|x\left[n\right]\right|}^{2}-{\left|x\left[n-L\right]\right|}^{2})/L.$ The initial $\hat{P}$ was approximated as the mean power of the signal used for the FFT computation, and the value of *L* was set to be long enough to reflect input amplitude changes [21].

### 2.2. Analysis Procedure

**Figure 1:**
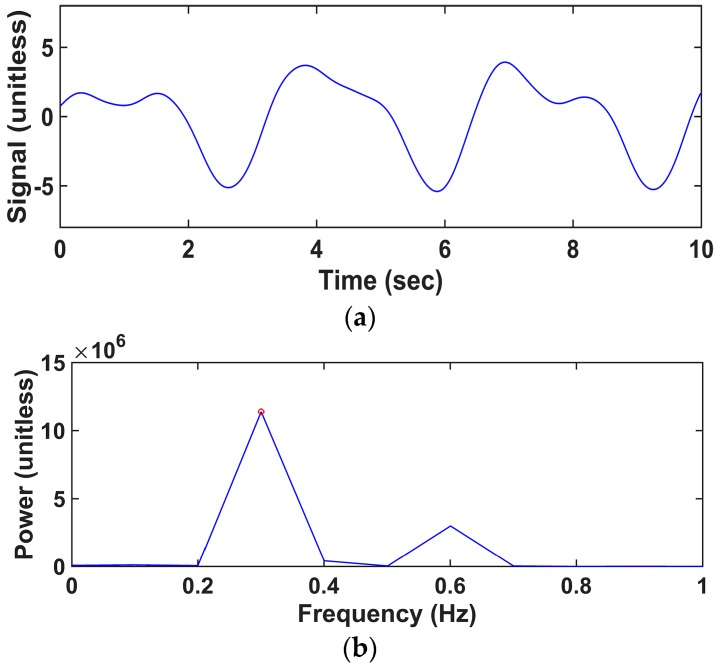
An example of a filtered photoplethysmogram (PPG) signal and its power spectrum: (**a**) PPG signal after filtering (unitless); (**b**) power spectrum (unitless) of (a). A hollow red circle represents a peak of which the power is maximum.

Before estimating RR from PPG, we first down-sampled the raw data in order to improve the computational efficiency. We then applied a third-order Butterworth high-pass filter with a cutoff frequency of 0.2 Hz and a low-pass filter with a cutoff frequency of 0.8 Hz to extract respiration-related components from PPG and to remove the DC offset component. These cutoff frequencies were determined considering a normal range of RR (12 to 30 breaths per minute) [15,19]. We estimated the initial *θ* using the first 10 s signal, and the value of *L* was set to time points corresponding to the 10 s signal (*L* = 1000) Figure 1a shows the bandpass-filtered signal, and Figure 1b shows the power spectrum of the bandpass-filtered signal. A frequency with the highest power between 0.2 and 0.8 Hz was determined to be the fundamental frequency, as marked with a red circle in Figure 1b. 

### 2.3. Offline Data for Validation

We validated our RR estimation method using a benchmark dataset called the CapnoBase [23], which can be downloaded from [24]. The dataset is composed of multiple physiological signals recorded from 29 children and 13 adults. The PPG signals were recorded at a sampling frequency of 300 Hz for 8 min. All of the data processing and statistical analyses were performed using MATLAB^®^ (Mathworks, Natick, MA, USA).

### 2.4. Performance Evaluation

The accuracy of the RR estimation was assessed using the root mean square error (RMSE) defined as

(5) $$\mathrm{RMSE}=\sqrt{\frac{1}{n}\overset{n}{\underset{i=1}{\sum }}{\left(x_{i}^{est}-x_{i}^{ref}\right)}^{2}}$$

where *n* is the total number of time samples, and $x^{est}$ and $x^{ref}$ represent the estimated and reference RR values, respectively. The reference RR values were linearly interpolated to calculate the RMSE value. We compared the RMSE values of AIIRE with those of ALRE. All parameters of both methods were optimized for the best accuracy. The Wilcoxon signed rank test was used for the statistical analyses after testing the Gaussianity using the Kolmogorov–Smirnov test [25]. In addition, to evaluate the performance of ALRE, a measure called a convergence time (CT) was introduced, which represents the first moment when the RMSE of ALRE becomes less than 1 breath/min for 1 s. In ALRE, a short CT is required in mobile applications, but it generally results in large errors after the CT [20]. Since CT can be controlled via two parameters of ALRE [20], changes in the RMSE value evaluated from the CT to the end of the data were evaluated with different CT values by changing the values of two parameters of ALRE.

### 2.5. Implementation of a Real-Time RR Estimation System

We implemented a PPG-based real-time RR estimation system using a biosignal recording system (ActiveTwo, BioSemi, Amsterdam, The Netherlands) with a PPG sensor. The PPG sensor is a commercialized finger clip-type sensor (MLT1020FC, ADI Instruments, Dunedin, New Zealand) that uses reflected infrared light to measure the changes in blood flow. The PPG signal was recorded at a sampling frequency of 2048 Hz. In the real-time RR estimation, we down-sampled the raw PPG signal from 2048 Hz to 128 Hz, and applied the proposed method for the real-time estimation of RR.

In the online experiment, a participant (male, 25 years old) was asked to take a deep breath following the flickering frequency of a black-gray circle (a respiratory pacemaker) displayed on the upper right corner of the computer monitor, when the current RR value was also displayed on the monitor.

## 3. Results

**Figure 2:**
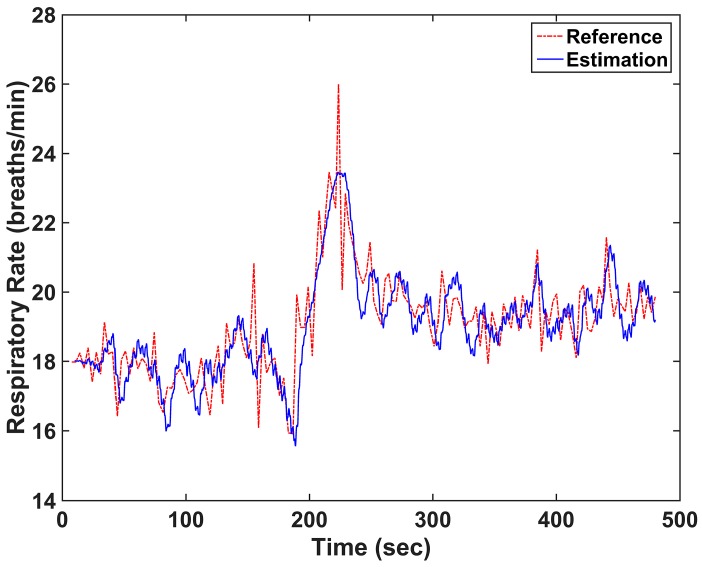
An example of a respiratory rate (RR) estimation of the 8 min data. The solid line is the RR estimated using the proposed method and the dash-dot line is the reference RR. The resultant root mean square error (RMSE) value was 0.7381 breaths/min.

An example of the RR estimation results in our offline simulation study is shown in Figure 2, where the solid line is the estimated RR and the dash–dot line is the reference RR recorded using capnography. In this figure, the RMSE value was 0.7381 breaths/min. The parameters in Equations (1)–(4) were optimized to minimize the RMSE value, when the values of *r* and the constant were 0.999, and 0.4 × 10^−8^, respectively.

**Figure 3:**
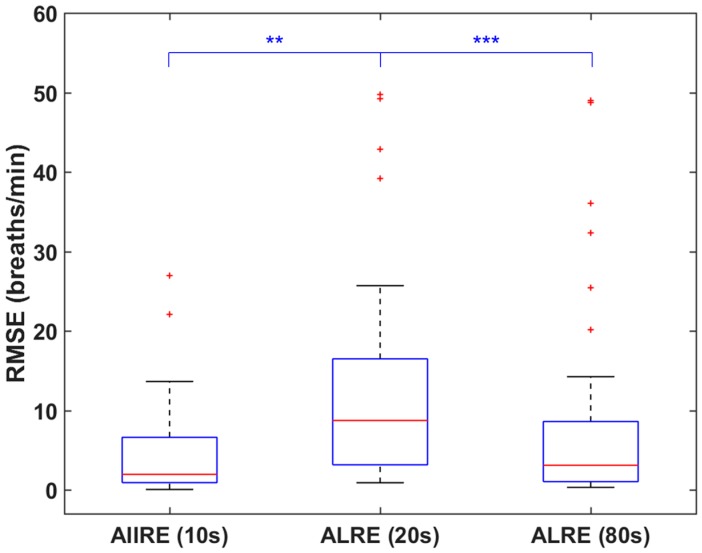
Distributions of root mean square errors (RMSEs) of respiratory rate (RR) estimation results when the proposed method and the adaptive lattice-type respiratory rate estimator (ALRE) were applied to the same dataset. Lower quartile, median, and upper quartile values are displayed in the box plots. Whiskers are used to represent the most extreme values within 1.5 times the interquartile range from the quartile. Outliers are displayed as crosses. ** (*p* < 0.01) and *** (*p* < 0.001) represent a statistically significant difference. AIIRE: Adaptive Infinite Impulse response filter-based Respiratory rate Estimator.

Figure 3 shows the distributions of RMSEs of RR estimation results when the proposed method and ALRE were applied to the same offline dataset. The median RMSE of the proposed method estimated from 20 s to the end of data was 1.95 breaths/min, and that of the ALRE was 8.62 breaths/min (CT of ALRE was set to 20 s). Note that AIIRE used only the 10 s data for initialization, but the RMSE was computed from the 20 s data, because some offline PPG data did not include reference signals before 20 s. The difference between the two results were statistically tested using the Wilcoxon signed rank test, as the two distributions did not pass the Kolmogorov–Smirnov test (*p* < 0.001). The statistical analysis showed that the two distributions are significantly different (*p* < 0.01), even though more time was given to ALRE than the proposed method. When we computed the RMSE of ALRE from 80 s to the end of data, the RMSE value was considerably reduced to 2.54 breaths/min, suggesting that the RR estimated using ALRE was converging to the reference RR. The distribution of the RMSE of ALRE then became comparable to that of the proposed method (*p* > 0.05), which demonstrates that ALRE requires a minimum of eight times as long as the proposed method to estimate RR as precisely.

**Figure 4:**
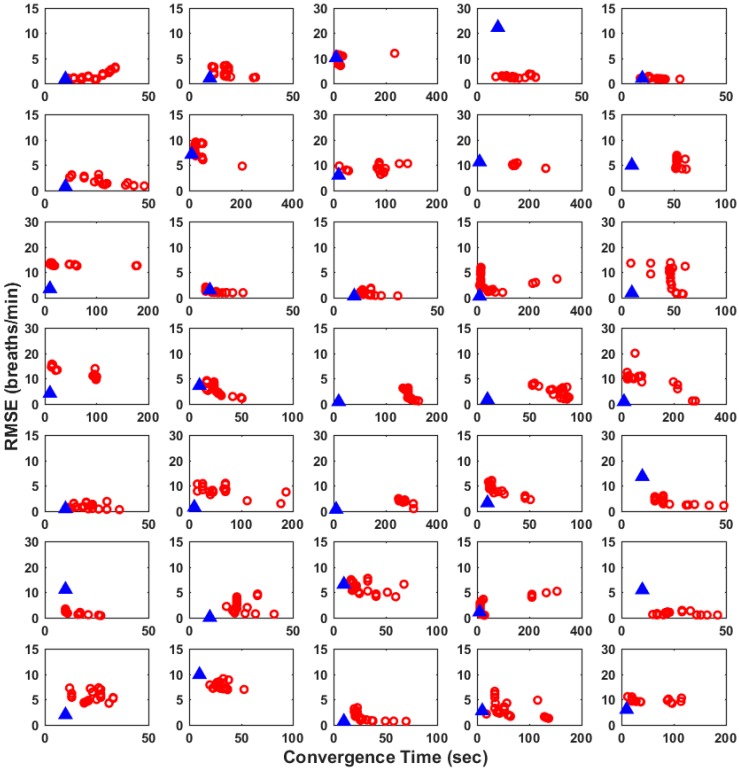
Scatter plots comparing root mean square errors (RMSEs) and convergence times (CTs) of the proposed method and adaptive lattice-type respiratory rate estimator (ALRE) in 35 PPG data. The solid blue triangle represents the RMSE and the time necessary for the initialization of the proposed method, while the hollow red circles represent 21 RMSEs and CTs of ALRE. Black arrows are marked only when the performance of ALRE was better than that of the proposed method.

As previously mentioned, the CT of ALRE can be adjusted by controlling two parameters of the ALRE algorithm. If the parameters are small, the convergence time will be shortened, but the performance (accuracy) is reduced [20]. In Figure 4, we scattered RMSEs of the proposed method and 21 RMSEs of ALRE when two parameters of ALRE were changed from 0.975 to 0.995. We used only 35 out of 42 data, because CT could not be evaluated at all in seven data, indicating that the RMSE of ALRE never dropped below 1 breath/min for the entire 8 min time period in the seven subjects’ data. 

In Figure 4, the blue filled triangle represents the RMSE and the time necessary for the initialization (10 s in this study) of the proposed method, while the red hollow circles represent 21 RMSEs and the CTs of ALRE. As can be seen from the graphs, the shorter distance between the origin and a scatter point indicates that the proposed method can estimate RR more quickly and accurately. In this regard, the proposed method showed better performance in 30 cases out of 35 data. The five cases when ALRE showed better performance than the proposed method are marked with a small black arrow inside each graph. The average of the shortest CT of all 35 cases was 29.49 s, and the average of the CT with the smallest RMSE was 84.23 s.

**Figure 5:**
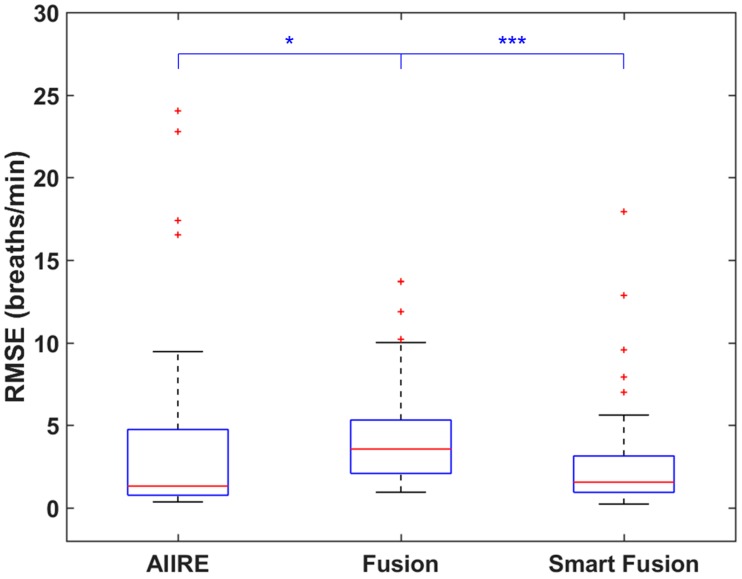
Distributions of root mean square errors (RMSEs) of respiratory rate (RR) estimation results when the proposed method and the smart fusion algorithms were applied to the same dataset. Lower quartile, median, and upper quartile values are displayed in the box plots. Whiskers are used to represent the most extreme values within 1.5 times the interquartile range from the quartile. Outliers are displayed as crosses. * (*p* < 0.05) and *** (*p* < 0.001) represent a statistically significant difference.

Figure 5 shows the distributions of the RMSE of AIIRE and two existing algorithms referred to as Fusion and Smart Fusion. The results of Fusion consisted of RRs estimated every 3 s starting from 16 s, and the results of Smart Fusion excluded RRs with large errors or RRs computed for windows with significant artifacts from the results of Fusion [19]. The cutoff frequency of AIIRE was changed to 4 to 48 breaths/min for fair comparison with the smart fusion algorithms. The median values of AIIRE, Fusion, and Smart Fusion were 1.33 breaths/min, 3.57 breaths/min, and 1.56 breaths/min, respectively. Since any of the three distributions did not pass the normality test, their differences were statistically tested using a Friedman test. The result of the Friedman test and the post-hoc analysis with Wilcoxon signed rank test showed a significant difference between Fusion and Smart Fusion (Bonferroni corrected *p* < 0.001) and between AIIRE and Fusion (*p* < 0.05), but did not exhibit significant differences between AIIRE and Smart Fusion.

**Figure 6:**
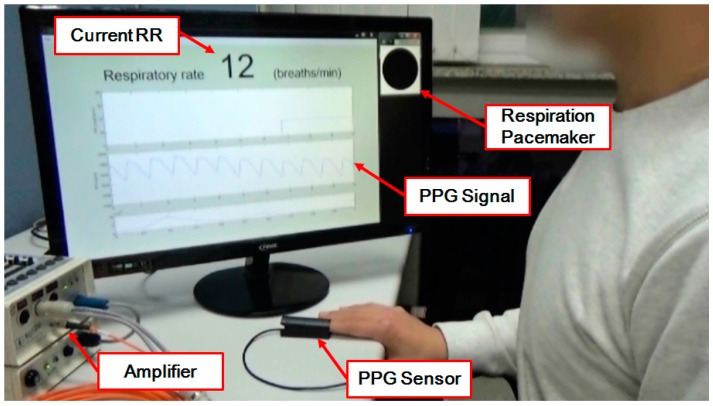
A screenshot of an online experiment that shows the detailed experimental conditions. A finger clip-type photoplethysmography (PPG) sensor was connected to a biosignal amplifier. Current respiratory rate (RR) value was displayed on the computer monitor while a study participant was taking a deep breath following the flickering frequency of a respiration pacemaker (a black-gray circle on the upper right corner of the monitor).

We also implemented an online RR estimation system which demonstrated the practical applicability of the proposed method in real-time RR estimation. Figure 6 shows a screenshot of the online experiment. In this experiment, we seamlessly monitored whether the estimated RR quickly caught up to the target RR that was randomly changing over time. The flickering frequency of the respiratory pacemaker on the screen guided the study participant’s respiration periods. The movie file is attached to this manuscript as a supplementary multimedia file (Video S1), and can also be found on [26].

## 4. Discussions and Conclusions

In this study, a new method for the fast and reliable real-time estimation of RR from PPG was proposed. We adopted the adaptive IIR notch filter, which has not yet been applied to the PPG-based estimation of RR. In general, it is known that adaptive IIR notch filters are unstable, may converge to local minima, and have a slower convergence rate than the adaptive finite impulse response (FIR) notch filters [27], although the IIR filters are much less computationally demanding than the FIR filters. However, the adaptive notch filter used in this study was not only free from the multiple minima problem because it is a single-notch type filter [21,22], but also has a fast convergence rate by using the normalized LMS algorithm adopting variable step-size *μ*[*n*] [27]. In addition, we adopted a second-order IIR notch filter because it is more efficient than third or fourth-order IIR notch filters. This is because it has a fewer number of adjustable coefficients than higher-order filters [22]. In the normalized LMS algorithm, we updated the local input signal power using just two points to reduce the overall computational burden. This form is obviously more cost effective than the general sliding window form. A recursive form using just one point is also more cost effective than the sliding window form, but the form using two points is more efficient because several parameters should be adjusted experimentally when using the recursive form.

**Figure 7:**
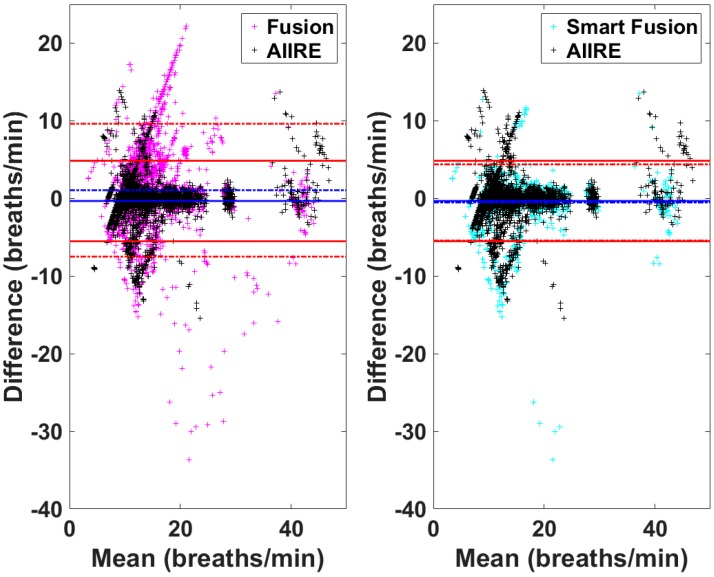
Bland–Altman plots comparing estimation errors of Adaptive Infinite Impulse response filter-based Respiratory rate Estimator (AIIRE) and two fusion algorithms (Fusion and Smart Fusion). The solid lines represent mean ±1.96 s.d. of the proposed method (AIIRE), and the dash–dot lines those of Fusion and Smart Fusion.

The proposed method, AIIRE, was applied to an offline dataset composed of 42 PPG data, and the performance was compared with that of the conventional approach, ALRE. Our simulation results show that the proposed method estimates RR more accurately than ALRE, even with a much shorter initialization time. In addition, we compared the performance of AIIRE with those of smart fusion algorithms referred to as Fusion and Smart Fusion. The comparison of RMSE among three methods showed that AIIRE can estimate RR more accurately than Fusion and Smart Fusion, although the difference between AIIRE and Smart fusion was not statistically significant. In order to further investigate the characteristics of three methods, we plotted the Bland–Altman plots of three results obtained using three different methods [28] in Figure 7, where the differences between the continuously estimated RR and gold standard RR are scattered (the outlier data in Figure 5 were excluded). As readily seen from the figures, the standard deviation of AIIRE is smaller than that of Fusion and larger than that of Smart Fusion, but both smart fusion algorithms showed many discrete points with much larger errors than AIIRE. Although the Smart Fusion method yielded better RR estimates in terms of the overall accuracy, it needs to be noted that the estimation of RR in Smart Fusion might not be continuous along time, because Smart Fusion excludes RR with large error or RR estimated from a window with significant artifacts. In addition, Smart Fusion requires a longer initialization time (16 s) than AIIRE (10 s). Considering all these factors, it is expected that the proposed method can be a promising alternative of the conventional Smart Fusion for the real-time “seamless” estimation of RR in practical applications. 

In healthcare applications using wearable or mobile devices, the fast and reliable estimation of RR is of great value [19]. Although we did not directly compare the accuracies of the RR estimation among all the existing algorithms, the proposed method has a clear advantage over many of the other methods in that it does not require the use of overlapping moving windows, which enables the continuous estimation of RR with less computational cost. This characteristic might be specifically useful in mobile healthcare applications for which computing power is generally limited.

Although the scope of this study did not include the sensing of PPG with wearable devices, they generally suffer from large physiological and environmental noise/artifacts. Since the estimated RR can also be affected by the contaminated PPG signals, the development of new methods for the robust estimation of RR in noisy environments is necessary; e.g., use of an adaptive Comb filter [29] for the estimation of RR after enhancing the respiration related signals—this is an avenue we would like to pursue in future studies.

## Supplementary Materials

The following are available online at http://www.mdpi.com/1424-8220/16/9/1494/s1, Video S1: Real-time Respiratory Rate Estimation system.
